# Stroke risk and mid-term survival by proximal extent of endovascular aortic repair

**DOI:** 10.1093/bjs/znag008

**Published:** 2026-02-07

**Authors:** Gisli Jonsson, Kevin Mani, Anders Wanhainen, David Lindström

**Affiliations:** Division of Vascular Surgery, Department of Surgical Sciences, Uppsala University, Uppsala, Sweden; Division of Vascular Surgery, Department of Surgical Sciences, Uppsala University, Uppsala, Sweden; Division of Vascular Surgery, Department of Surgical Sciences, Uppsala University, Uppsala, Sweden; Department of Surgical and Perioperative Sciences, Umeå University, Umeå, Sweden; Division of Vascular Surgery, Department of Surgical Sciences, Uppsala University, Uppsala, Sweden; Department of Clinical Science and Education, Karolinska Institutet, Division of Vascular Surgery, Södersjukhuset, Stockholm, Sweden

## Abstract

**Background:**

Recent advancements in endovascular aortic repair (EVAR) have expanded treatment to increasingly proximal aortic segments, including the thoracic and thoracoabdominal aorta as well as the arch. Coherent real-world data on stroke risk after EVAR, stratified by the proximal landing zone, and the impact of perioperative stroke on mid-term survival are scarce.

**Methods:**

A population-based multiregistry retrospective analysis was performed on prospectively collected data for all EVARs performed in Sweden between 15 May 2018 and 15 May 2023. Data were collected from the Swedish Vascular Registry (Swedvasc) and cross-referenced with the Swedish Patient Registry, the Swedish Cause of Death Registry, and the Swedish Stroke Registry. The effect of perioperative stroke on mid-term survival was analysed and the relationship between the incidence of stroke and the proximal landing zone was analysed.

**Results:**

Some 4842 EVARs were performed. Perioperative stroke occurred in 1.6% (79 of 4842). Stroke risk was associated with the proximal landing zone, with a stroke rate of 22% in zone 0. In multivariable analysis, the OR for stroke in zone 0 was 41.58 (95% c.i. 18.46 to 93.70); *P* < 0.001. The 30-day mortality rate was 24% (19 of 79) and 3.5% (169 of 4763) for patients with and without perioperative stroke respectively. This difference persisted over time, with a 4-year survival probability of 48.6% and 72.5% for patients with and without stroke respectively (HR 2.17 (95% c.i. 1.26 to 3.72); *P* < 0.001).

**Conclusion:**

Stroke is strongly associated with the proximal landing zone, being more likely when landing proximal to the supra-aortic vessels. Perioperative stroke impacts patient survival and should be taken into consideration during patient selection, and efforts should be made to reduce the risk of stroke during proximal repair.

## Teaser

This was a population-based multiregistry study of all endovascular aortic repairs (EVARs) performed in Sweden between 15 May 2018 and 15 May 2023. The aim was to analyse the risk of perioperative stroke in relation to the proximal aortic landing zone and to analyse the effect of perioperative stroke on mid-term survival. Out of 4842 patients who underwent EVAR, 1.6% developed stroke, which was strongly associated with the proximal landing zone, with the highest risk of perioperative stroke associated with landing zone 0 (22%). Additionally, perioperative stroke affected the mid-term survival of patients, with an HR of 2.17. This should be accounted for when planning proximal EVAR.

## Introduction

The risk of stroke with surgery and general anaesthesia is well documented^1^ and that risk is especially relevant in endovascular aortic repair (EVAR). The risk of stroke with aortic intervention is affected by multiple factors, such as age, sex, duration of surgery, atherosclerosis in the aortic arch, involvement of the supra-aortic vessels, and upper extremity access during the procedure^2^. In the literature, the risk of stroke varies, being as low as 0.1–0.6%^3,4^ in infrarenal EVAR, 2.1–2.7% in thoracic endovascular aortic repair (TEVAR) without arch involvement^4,5^, and 11–17%^5–9^ in advanced aortic arch repair. In TEVAR procedures, coverage of the left subclavian artery (LSA) is associated with an increased risk of stroke, and LSA revascularization may or may not reduce that risk.^2,5,7,10–14^. Aneurysmal disease in the aortic arch is associated with an increased perioperative stroke risk in comparison with other thoracic aortic pathologies^8^.

Perioperative stroke occurring during EVAR is mainly of an embolic nature; thrombus, arteriosclerotic material, and air embolus^11,15^. Perioperative stroke can also be caused by haemodynamic instability resulting in cerebral hypoperfusion^12,16^. The short-term effect of perioperative stroke can be devastating, with 50% of patients suffering from disability and up to 25% of patients dying within 30 days^14,15^.

In recent years, with the availability of endovascular techniques for supra-aortic revascularization using branched or fenestrated devices, endovascular repair has increasingly been utilised in the aortic arch^17–20^. In the thoracoabdominal and paravisceral aorta, the availability of branched and fenestrated stentgrafts has resulted in an increasing use of endovascular techniques. Thus, the extension of EVAR is rapidly moving proximally. This involves increased arch manipulation, either with the stentgrafts during arch repair or with wires and catheters during thoracoabdominal/paravisceral repair. There are scarce real-world data on the risk of stroke in EVAR depending on the proximal landing zone. Mid-term survival after endovascular repair and the association between perioperative stroke and mid-term survival is not well studied.

The aim of this study was to assess the incidence of stroke after EVAR in relation to the proximal aortic landing zone and to investigate the impact of perioperative stroke on mid-term survival based on nationwide registry data.

## Methods

### Study design and data collection

This study involved the retrospective analysis of prospectively collected data for all patients who underwent EVAR with proximal aortic landing zones ranging from zone 0 to zone 11 for aneurysm or dissection in Sweden between 15 May 2018 and 15 May 2023, identified in the Swedish Vascular Registry (Swedvasc). Swedvasc is a national registry of all vascular procedures performed in Sweden, with high internal and external validity for aortic procedures^21,22^. Data are registered prospectively and include patients’ demographic data, co-morbidity data, procedural data, and short- and long-term outcome data. After identification of the study population through Swedvasc, data were cross-referenced with three other national registries (using unique personal identifiers): the Swedish Stroke Registry, the Swedish Patient Registry, and the Swedish Cause of Death Registry. The Swedish Patient Registry contains data on all inpatient and outpatient care, with registration of diagnoses and surgical procedures based on standardized coding, and was employed for verification of all strokes. The Swedish Cause of Death Registry was used for verification of all deaths and strokes leading to death. The Swedish Stroke Registry was utilized to classify the severity and date of each stroke as well as to determine whether a stroke was of haemorrhagic or ischaemic origin. Additionally, the Swedish Stroke Registry was used to detect preoperative strokes in the previous 5 years. The study was approved by the regional ethics committee (2022-06646-01).

### Patient demographics and procedural data

Age, sex, co-morbidities, and possible risk factors for developing stroke were recorded. Risk factor definitions from Swedvasc were: cardiac risk—prior myocardial infarction, angina, chronic heart failure, or prior cardiac intervention; chronic pulmonary disease—symptomatic chronic obstructive pulmonary disease, emphysema, or other chronic symptomatic pulmonary disease; prior cerebrovascular lesion (CVL)—prior cerebral infarction or haemorrhage; and chronic kidney disease—confirmed chronic decrease in renal function. Aortic pathologies included were dissections and aneurysms; localization of disease was also recorded. The urgency of a procedure was recorded as acute, subacute, or elective. A subacute procedure was defined as an intervention during the original admission without the need for immediate surgery. Aneurysms were further defined as asymptomatic, symptomatic, or ruptured. The proximal extent of an endovascular repair was defined by the proximal aortic landing zone of the procedure, as described elsewhere^23^. The revascularization of any thoracic or abdominal aortic branches was also recorded.

### Outcomes

The incidence of perioperative stroke in relation to the proximal landing zone of an aortic stentgraft was analysed and the association between perioperative stroke and mid-term survival was explored.

A perioperative stroke as an outcome event was defined as the presence of a stroke (ischaemic or haemorrhagic) registered in any of the four registries (Swedvasc, the Swedish Patient Registry, the Swedish Stroke Registry, or the Swedish Cause of Death Registry) within 30 days of the procedure date. A stroke was categorized as a minor stroke or a major stroke. A major stroke was defined as a stroke requiring rehabilitation, a stroke with a National Institutes of Health Stroke Scale score of at least eight prospectively recorded in the Swedish Stroke Registry, a stroke with classification as a major stroke in Swedvasc, or a stroke registered as the leading cause of death. Transient ischaemic attacks were excluded from the analysis.

### Statistical analysis

Descriptive statistics are used for baseline data. Continuous variables are reported as mean(s.d.) or median (interquartile range (i.q.r.)), depending on normality, while categorical variables are reported as % (*n*). Normality was assessed using visual assessment of quantile–quantile plots and the Shapiro–Wilk test when appropriate, depending on sample size. Comparison between groups was performed using Pearson’s chi-squared test or Fisher’s exact test for categorical variables and the Mann–Whitney *U* test was used to compare ordinal variables between two groups. The primary outcome, stroke within 30 days, is reported as % (*n*) for each aortic landing zone. Age was categorized into three groups: ≤70, 71–79, and ≥80 years. Univariable analysis was performed on known and clinically relevant risk factors for stroke (proximal landing zone, age, sex, urgency of procedure, prior CVL, smoking status, hypertension, cardiac risk, diabetes, renal insufficiency, and aneurysm/dissection).

Factors with *P* < 0.200 were subsequently analysed in a multivariable regression analysis; no variables with *P* > 0.200 were deemed so clinically important as to warrant a forced inclusion in the multivariable model. Goodness-of-fit was assessed using −2 log likelihood for further refinement of the final model. Revascularization of thoracic and abdominal branches was not included in the multivariable regression model due to collinearity with the proximal landing zone variable. Revascularization of branches was instead analysed separately for respective landing zones. Due to a low number of events, proximal landings in zones 6–11 were grouped together and used as the reference for proximal landing zone in the multivariable analysis.

Kaplan–Meier analysis and a multivariable Cox regression model were utilized to explore the impact of perioperative stroke on mid-term survival. Univariable analysis was performed on clinically relevant and previously described co-morbidities in relation to mortality; factors with *P* < 0.200 were included in the Cox regression. Factors adjusted for in the Cox regression model were: age, sex, cardiac risk, chronic pulmonary disease, renal insufficiency, smoking, aortic pathology (dissection/aneurysm), perioperative stroke, urgency of procedure, and proximal aortic landing zone. A landmark analysis was also performed excluding events within the first 90 days after surgery. *P* < 0.050 was considered statistically significant. Statistical analysis was performed using SPSS^®^ (IBM, Armonk, NY, USA; version 28).

## Results

### Patient demographics

A total of 4842 patients underwent EVAR during the time interval, with a mean(s.d.) age of 75(8.3) years; 78.1% (3780) were male, 93.2% (4512) were treated due to an aneurysm, and 6.8% (330) were treated due to a dissection. See *Table 1*. Most procedures were elective (76.1% (3685)); the other procedures were acute (18.4% (890)) or subacute (5.5% (267)). See *Table 2*.

**Table 1:** Demographic characteristics of all patients who underwent EVAR in Sweden between 15 May 2018 and 15 May 2023 (overall and based on the proximal aortic landing zone)

Demographics	Total (*n* = 4842)	Zone 0 (*n* = 59)	Zone 1 (*n* = 39)	Zone 2 (*n* = 190)	Zone 3 (*n* = 248)	Zone 4 (*n* = 270)	Zone 5 (*n* = 627)	Zones 6–11 (*n* = 3409)
Age (years), mean(s.d.)	74.7(8.3)	73.1(6.7)	73.3(7.9)	67.7(13.7)	68.8(11.4)	70.7(11.6)	74.1(6.8)	76.1(7.0)
Male	78.1 (3780)	56 (33)	64 (25)	64.7 (123)	61.7 (153)	61.5 (166)	74.8 (469)	82.1 (2799)
**Smoking**								
Active	17.2 (832)	9 (4)	32 (7)	27.8 (25)	31.4 (49)	31.8 (55)	26.8 (130)	16.5 (559)
Prior	44.8 (2168)	61 (26)	59 (13)	43.3 (39)	47.4 (74)	46.2 (80)	60 (291)	48.0 (1637)
Never	14.3 (694)	30 (13)	9 (2)	28.9 (26)	21.2 (33)	22 (38)	13.2 (64)	15.1 (515)
Cardiac risk	41.7 (2017)	54 (32)	36 (14)	23.2 (43)	32 (77)	36.7 (98)	42 (261)	33.7 (1487)
Hypertension	79.4 (3843)	91 (52)	85 (33)	76.2 (138)	78.2 (190)	84.7 (221)	80.7 (494)	79.3 (2703)
Diabetes mellitus	15.2 (738)	7 (4)	5 (2)	7.6 (14)	12 (29)	13.4 (36)	12.7 (79)	16.8 (572)
Chronic pulmonary disease	26.8 (1293)	15 (9)	21 (8)	19 (35)	26.8 (64)	28.1 (74)	34.9 (216)	25.9 (882)
Chronic kidney disease	14.3 (964)	12 (7)	8 (3)	12.6 (23)	11.3 (27)	12.8 (34)	18.3 (113)	14.2 (485)
Dialysis	0.8 (41)	0 (0)	3 (1)	2.1 (4)	1.6 (4)	0.4 (1)	0.6 (4)	0.8 (26)
Prior aortic surgery	15.8 (763)	64 (38)	23 (9)	28.9 (55)	39.1 (97)	48.9 (132)	31.4 (197)	6.8 (233)
Prior CVL	15.9 (771)	22 (13)	18 (7)	11.1 (21)	14.1 (35)	17.8 (48)	15.9 (100)	16.0 (545)
**Anatomy**								
Aneurysm	93.2 (4512)	61 (36)	64 (25)	46.3 (88)	61.7 (153)	80 (216)	97.1 (609)	98.8 (3368)
Diameter (mm), mean(s.d.)	60.5(13.5)	58.2(12.9)	56.4(14.8)	45.5(13.8)	47.8(14.1)	52.8(16.3)	64.9(12.4)	50.4(17)
Symptomatic	8 (359)	17 (6)	8 (2)	19.3 (17)	13.1 (20)	13.4 (29)	6.6 (40)	7.2 (245)
Dissection	6.8 (330)	39 (23)	36 (14)	53.7 (102)	38.3 (95)	20 (54)	2.9 (18)	0.7 (24)
Rupture (aneurysm and dissection)	12.2 (552)	3 (2)	10 (4)	27.4 (52)	16.5 (41)	11.1 (30)	4.5 (28)	12.9 (439)

Data are % (*n*) unless otherwise indicated. Cardiac risk corresponds to prior myocardial infarction, angina, chronic heart failure, or prior cardiac intervention. Chronic pulmonary disease corresponds to symptomatic chronic obstructive pulmonary disease, emphysema, or other chronic symptomatic pulmonary disease. Prior CVL corresponds to prior cerebral infarction or haemorrhage. Chronic kidney disease corresponds to confirmed chronic decrease in renal function. EVAR, endovascular aortic repair; CVL, cerebrovascular lesion.

**Table 2:** **Perioperative (30-day) stroke, mortality, and complication rates after all EVARs in Sweden between 15 May 2018 and 15 May 2023** (**overall and based on urgency of procedure)**

Variable	Total (*n* = 4842)	Elective (*n* = 3685)	Subacute (*n* = 267)	Acute (*n* = 890)	P*
**Total infarction**	1.2 (60)	0.9 (32)	2.3 (6)	2.5 (22)	<0.001
Minor stroke	0.9 (42)	0.6 (23)	1.9 (5)	1.6 (14)	0.005
Major stroke	0.3 (16)	0.2 (7)	0.4 (1)	0.9 (8)	0.007
Retinal stroke	<0.1 (2)	0.1 (2)	0.0 (0)	0.0 (0)	1.000
Intracranial haemorrhage	0.4 (19)	0.2 (9)	0.7 (2)	0.9 (8)	0.011
Mortality	3.9 (188)	1.4 (53)	4.5 (12)	13.8 (123)	<0.001
Acute myocardial infarction	0.7 (33)	0.5 (19)	1.5 (4)	1.1 (10)	0.033
Kidney injury	3.0 (145)	1.8 (67)	3.4 (9)	7.8 (69)	<0.001
Bowel ischaemia	1.0 (47)	0.6 (22)	1.5 (4)	2.4 (21)	<0.001
Bowel resection	0.7 (32)	0.4 (15)	0.4 (1)	1.8 (16)	<0.001
Amputation of lower limb	<0.1 (4)	<0.1 (3)	0.0 (0)	0.1 (1)	0.670
Multiorgan failure	1.8 (86)	0.7 (26)	2.6 (7)	6.0 (53)	<0.001
**Spinal cord ischaemia**	1.8 (86)	1.4 (50)	1.9 (5)	3.5 (31)	<0.001
Transient	1.2 (57)	1.0 (36)	1.5 (4)	1.9 (17)	0.053
Permanent	0.6 (29)	0.4 (14)	0.4 (1)	1.6 (14)	<0.001

Data are % (*n*). *Chi-squared. EVARs, endovascular aortic repairs.

### Thirty-day outcome

The 30-day mortality rate was 1.4% (53), 4.5% (12), and 13.8% (123) in the elective group, the subacute group, and the acute group respectively (*P* < 0.001). See *Table 2*. The 30-day mortality rate was 24% (19) for patients with perioperative stroke and 3.5% (169) for patients without perioperative stroke (*P* < 0.001). Of 19 deaths in the stroke group, 8 (42%) were primarily caused by the neurological event.

### Overall stroke outcome

**Fig. 1:**
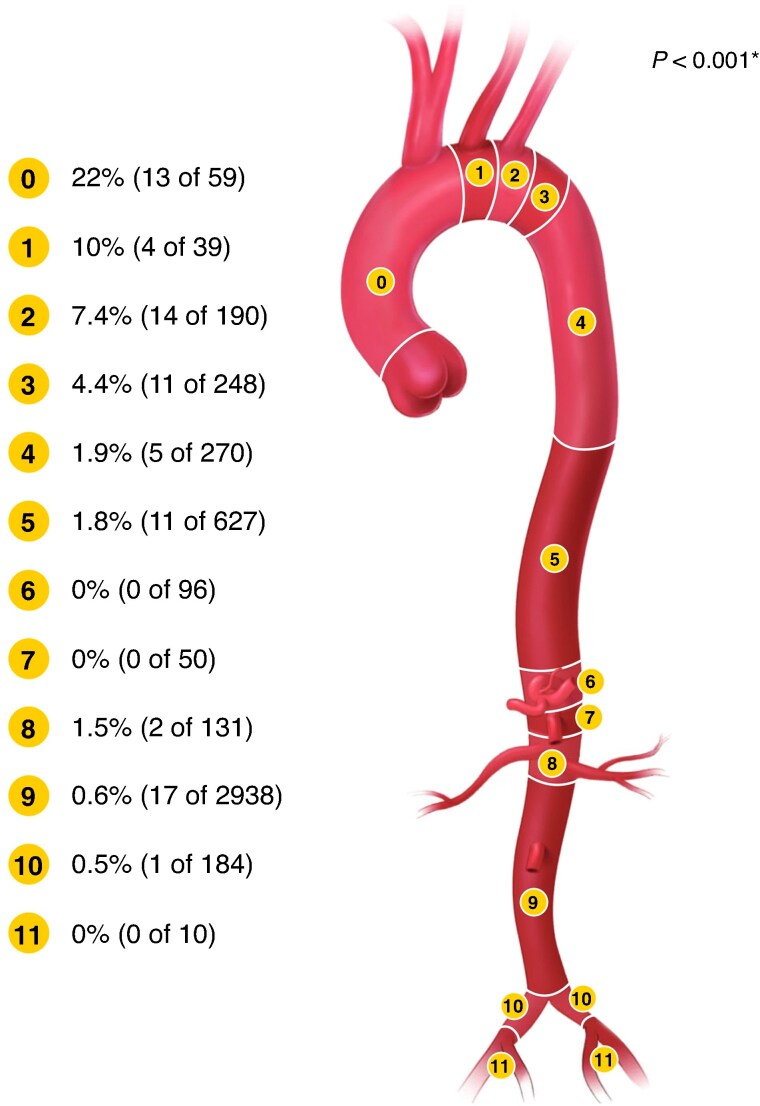
Total stroke incidence by proximal aortic landing zone *Chi-squared.

The stroke rate within 30 days was 1.6% (79), of which 58% (46) were minor, 39% (31) were major, and 3% (2) were retinal strokes. There was a significant association between the proximal landing zone and the 30-day stroke risk; the highest risk was associated with zone 0 (stroke rate of 22% (13 of 59)), followed by zone 1 (stroke rate of 10% (4 of 39)) and zone 2 (stroke rate of 7.4% (14 of 190)), and landing zones 3–11 had stroke rates that ranged from 0% to 4.4% (*Fig. 1*). Perioperative stroke risk for zone 0 and zone 1 was high for both acute patients (27% (3) and 11% (1) respectively) and elective patients (23% (10) and 10% (3) respectively). See Table 3. Amongst the strokes, 24% (19) were haemorrhagic, occurring most often in the more proximal landings (zone 0 (2% (1 of 59)), zone 1 (3% (1 of 39)), zone 2 (2.1% (4 of 190)), and zone 3 (2.0% (5 of 248))); the risk was 0–1.0% in landings distal to zone 3 (*P* < 0.001). In the more distal landing zones, the occurrence of haemorrhagic stroke was most common in landing zone 5 and zone 6 (0.5% (3 of 627) and 1% (1 of 96) respectively) while infrarenal EVAR (zone 9) had a 0.1% (3 of 2920) risk of haemorrhagic stroke. Major strokes were most common in zone 0 and zone 1 (7% (4 of 59) and 5% (2 of 39) respectively). In zone 0, 38 patients had undergone a prior aortic repair, of whom 87% (33) had undergone ascending aortic graft repair. When compared with landing in a native ascending aorta there was no difference in stroke rate (21% (7 of 33) *versus* 23% (6 of 26); *P* = 0.864). Stroke risk was proportionally higher for patients treated due to dissections compared with aneurysms (5.5% (18) and 1.4% (61) respectively; *P* < 0.001). Although, for procedures only involving the supra-aortic trunks (proximal landing zones 0–2), 13.4% (20) of aneurysm patients had a stroke and 7.9% (11) of dissection patients had a stroke (*P* = 0.182). In arch repair, the brachiocephalic trunk was most commonly revascularized using a branch (64% (38)), then fenestration (5% (3)), *in situ* laser fenestration (ISLF) (2% (1)), scallop (2% (1)), and reimplantation (2% (1)); there was no statistical difference in stroke risk between the different revascularization methods (*P* = 0.362). Revascularization of the left carotid artery was most commonly performed using a branch (66% (39)), then bypass (9% (5)), fenestration (5% (3)), ISLF (2% (1)), scallop (2% (1)), and reimplantation (2% (1)); there was no statistical difference in stroke risk between the different revascularization methods (*P* = 0.811). In procedures involving proximal landing zone 2, the subclavian artery was revascularized in 29.5% (56), most commonly using fenestration (28), then ISLF (12), bypass (8), chimney (6), and scallop (2). The rate of stroke was 3.6% (2) with subclavian revascularization and 9.0% (12) without subclavian revascularization (*P* = 0.239). Stroke risk in complex EVAR procedures involving abdominal branch revascularization was 1.9% (19), which was higher than in standard infrarenal EVAR with landing in aortic zone 9 (0.6% (16)); *P* < 0.001.

**Table 3:** Stroke risk based on operative urgency, disease pathology and the proximal aortic landing zone for all EVAR in Sweden between 15 May 2018 and 15 May 2023

	Total (*n* = 4842)	Zone 0 (*n* = 59)	Zone 1 (*n* = 39)	Zone 2 (*n* = 190)	Zone 3 (*n* = 248)	Zone 4 (*n* = 270)	Zone 5 (*n* = 627)	Zones 6–11 (*n* = 3409)
**Pathology**								
Aneurysm	1.4 (61)	25 (9)	16 (4)	8 (7)	3.9 (6)	1.9 (4)	1.8 (11)	0.6 (20)
Dissection	5.5 (18)	17 (4)	0 (0)	6.9 (7)	5.3 (5)	1.9 (1)	0.0 (0)	4.3 (1)
**Urgency**								
Acute	3.4 (30)	27 (3)	11 (1)	11.2 (11)	4.3 (4)	3.1 (2)	4.8 (11)	1.2 (7)
Subacute	3.0 (8)	0 (0)	0 (0)	11.8 (2)	11.1 (3)	0 (0)	3 (1)	1.2 (2)
Elective	1.1 (41)	23 (10)	10 (3)	1.3 (1)	3.1 (4)	1.6 (3)	1.4 (8)	0.5 (12)

Data are % (*n*). EVAR, endovascular aortic repair.

### Logistic regression analysis

After univariable analysis of risk factors for perioperative stroke, age, proximal landing zone, urgency of procedure, sex, and prior CVL were included in the multivariable analysis (*Table S1*). Proximal landing in aortic zones 0–3 and 5 (compared with proximal landing in aortic zones ≥6), age between 71 and 79 years, prior CVL, and acute urgency were independent risk factors for perioperative stroke.

### Mid-term survival

**Fig. 2:**
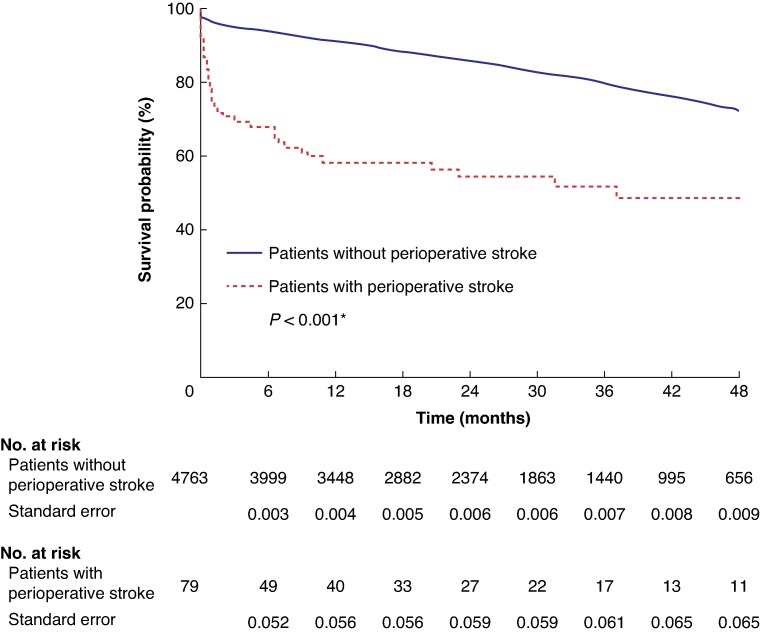
Kaplan–Meier survival analysis for the effect of perioperative stroke on mid-term survival *Log rank.

The median follow-up was 24 (i.q.r. 11–39) months. Survival probability in the absence and presence of perioperative stroke is shown in *Fig. 2*. One-year survival probability was 91.1% (95% c.i. 90.0% to 92.0%) for patients without stroke and 58.2% (95% c.i. 47.1% to 69.3%) for patients with stroke. Four-year survival probability was 72.5% (95% c.i. 70.5% to 74.5%) for patients without stroke and 48.6% (95% c.i. 35.7% to 61.5%) for patients with stroke. In a Cox regression analysis, perioperative stroke had a significant effect on mid-term survival, with an HR of 2.17 (95% c.i. 1.26 to 3.72) (*Table S2*). Removing events within the first 90 days after surgery in a landmark analysis did not change this; those with stroke still had poorer survival (*P* = 0.010, log rank) (*Fig. S1*). A subgroup analysis demonstrated significantly decreased 21-month survival in the major stroke group compared with the minor stroke group (*Fig. S2*).

## Discussion

In this nationwide population-based multiregistry analysis, the risk of stroke was significantly associated with the proximal aortic landing zone. The risk of stroke increased from 0% in the most distal landing zone to 22% in zone 0. This finding underlines the need for measures to reduce stroke risk, especially in proximal EVAR procedures, including potential for improved patient selection as well as operative technique.

The present data are consistent with a prior meta-analysis^5^, which demonstrated a similar association between the proximal landing zone and perioperative stroke, although it only included TEVAR patients. There are also two registry-based studies that analysed stroke risk related to landing zone and repair technique, but one excluded arch fenestrated devices^4^ and the other^8^ only analysed patients undergoing TEVAR; these studies did not explore mid-term survival. The high stroke rate when landing in zone 0 remained high when only analysing elective procedures (23% (10)); other studies have demonstrated a high stroke risk when landing in zone 0, although somewhat lower (6.3–17.5%)^4,8,9^. The reason for lower stroke rates could partly be explained by different case mixes (for example combining landing zone 0 and zone 1, excluding emergency procedures, and including thoracoabdominal aneurysms). Another explanation to consider is that, in the literature, there is a high incidence of silent brain infarction, ranging from 21% to 68%, after TEVAR^24–27^. Additionally, the present study was a multiregistry study that included data on stroke incidence from four different registries, minimizing the risk of detection bias. Additional strokes were identified in the national stroke registry and the national patient registry that were not encountered in Swedvasc. No additional strokes were identified in the cause of death registry; see *Fig. S3*. The risk of perioperative stroke in thoracic endovascular repair could have been previously under-reported.

In comparison with endovascular repair, a meta-analysis on frozen elephant trunk (FET) procedures demonstrated a pooled stroke risk of 7.6%^28^. Another study explored stroke outcome in FET depending on operative indication and demonstrated a higher total disabling stroke rate of 16% (8)^29^. None of the patients in the present study was operated on using FET procedures and direct comparison of endovascular arch repair with FET procedures should not be done, as patients undergoing endovascular arch repair are often deemed unfit for open repair.

Overall, the risk of stroke was proportionally higher for dissection patients compared with aneurysm patients (5.5% *versus* 1.4%), but this risk got reversed for procedures involving proximal landing zones 0–2, with a tendency towards higher stroke risk for patients with aneurysms compared with dissections (13.4% and 7.9% respectively; *P* = 0.182). This is in accordance with prior publications that have demonstrated a higher stroke risk in aneurysmatic disease when the aortic arch is involved^9,17^.

Regarding revascularization of the LSA in zone 2; ‘no revascularization’ was associated with a higher stroke risk compared with revascularization, although this was not significant (9.0% *versus* 3.6%; *P* = 0.239). The lack of a significant difference for LSA revascularization in the present study could be explained by a type II error.

In addition to direct involvement of supra-aortic vessels, simply traversing these vessels with guidewires and devices is also a risk factor for developing perioperative stroke^2^. Generally, in Sweden, the use of arm or neck access has diminished in favour of groin access with steerable sheaths since 2018. Unfortunately, information on arm or neck access is not available in Swedvasc. Perioperative anticoagulation is also of importance to decrease the risk of thrombosis and embolism^30,31^. Surgeons often aim for a longer activated clotting time (ACT) in arch repair compared with abdominal aortic repair; also, in abdominal repair, the ACT is traditionally longer for fenestrated EVAR/branched EVAR compared with infrarenal EVAR. In the present study, 24% (19 of 79) of the strokes were of haemorrhagic origin and proportionally occurred most often in arch repair (2–3%) and thereafter in more complex fenestrated EVAR/branched EVAR abdominal repair involving proximal landing in zone 5 and zone 6 (0.5–1%). This could indicate that the longer ACTs aimed for in arch repair and in fenestrated EVAR/branched EVAR actually increase the stroke risk rather than decrease it. Unfortunately, there were no data on ACTs in the present study for analysis.

The present study demonstrated that perioperative stroke had a high impact on the 30-day survival; the mortality rate was 24% and 3.5% for patients with and without perioperative stroke respectively. In addition, perioperative stroke had a significant effect on mid-term survival, with a 1-year survival probability of 58.2% and 91.1% for patients with and without perioperative stroke respectively. Furthermore, the effect of perioperative stroke on survival persisted even after a landmark analysis where all deaths within the first 90 days after surgery were excluded.

The stroke rate and associated poor survival reported in the present study need to be taken into consideration during patient selection and when obtaining consent for endovascular aortic procedures involving proximal landing in the aortic arch. A total stroke rate of 0.6% for infrarenal EVAR is an acceptable risk; the risk increases to 1.9% for more complex fenestrated EVAR/branched EVAR. This is possibly due to more proximal guidewire placement, longer procedures, and more extensive aortic disease.

The present study has some important limitations. First, data collection was retrospective and registry-based, which means that the variables that were used were limited to those available in the registries. Another limitation was the heterogeneity of the population-based cohort; disease pathophysiology, urgency of procedure, and patient demographics all varied. Additionally, even though the overall cohort was large, the number of procedures involving the most proximal aortic zones was low and, for some subgroups, the risk of a type II error was evident. Finally, there were limitations regarding the Swedvasc database, namely the lack of detailed data on anatomical factors that could aid in the interpretation of stroke risk, such as the type of aortic arch, the volume of the aneurysmal thrombus lining, and the stenosis grade of the aortic branches. Another possibly important missing variable was preoperative treatment with antithrombotics.

## Supplementary Material

znag008_Supplementary_Data

## Data Availability

Data are available upon reasonable request.
