# Supplementary material for: Stroke risk and mid-term survival by proximal extent of endovascular aortic repair
Source: Br J Surg. 2026 Feb 7;113(3):znag008. doi: 10.1093/bjs/znag008 (PMC13017605; doi:10.1093/bjs/znag008)
Supplement: znag008_Supplementary_Data [file znag008_supplementary_data.docx]

**Stroke risk and mid-term survival by proximal extent of endovascular aortic repair – a population-based registry study**

Jonsson G, MD PhD^1^, Mani K, MD PhD^1^, Wanhainen A, MD PhD^1,2^, Lindström D, MD PhD^1,3^

1. Division of Vascular Surgery, Department of Surgical Sciences, Uppsala University, Uppsala, Sweden.

2. Department of Diagnostics and Intervention, Surgery, Umeå University, Umeå, Sweden

3. Division of Vascular surgery, Dep of Clinical science and Education, Karolinska Institutet, Södersjukhuset, Stockholm, Sweden

**Corresponding author:**

**Name: Gísli Gunnar Jónsson**

**Email:** [**gisli.jonsson@uu.se**](mailto:gisli.jonsson@uu.se)

**Adress: Sjukhusvägen, 75185, Uppsala, Sweden**

| **Supplementary Figures and Tables** |  |
| --- | --- |
| **Supplementary Figure 1. Venn diagram of distribution of strokes**  **amongst registries.**  *A. Swedvasc, B. The national stroke registry, C. The National Patient registry*  *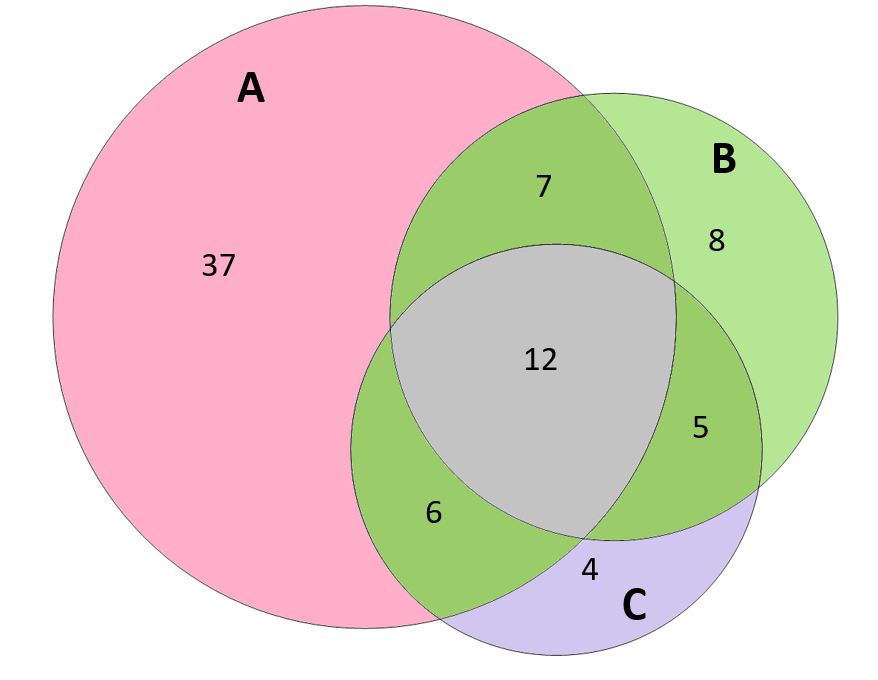* | *Page7* |
| **Supplementary figure 2. Kaplan-Meier landmark analysis on the effect of perioperative stroke on mid-term survival. Survival analysis starts 90 days post-operatively and deaths before that time period have been excluded. *Log rank**  **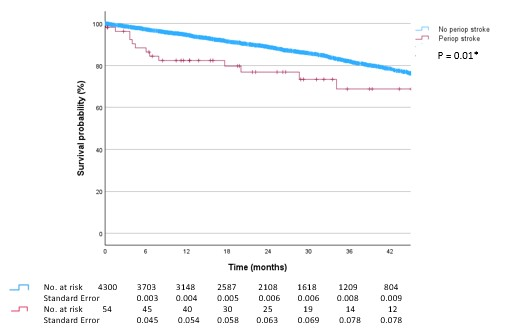**  **Supplementary Figure 3. Kaplan-Meier subgroup survival analysis on major and minor stroke**  **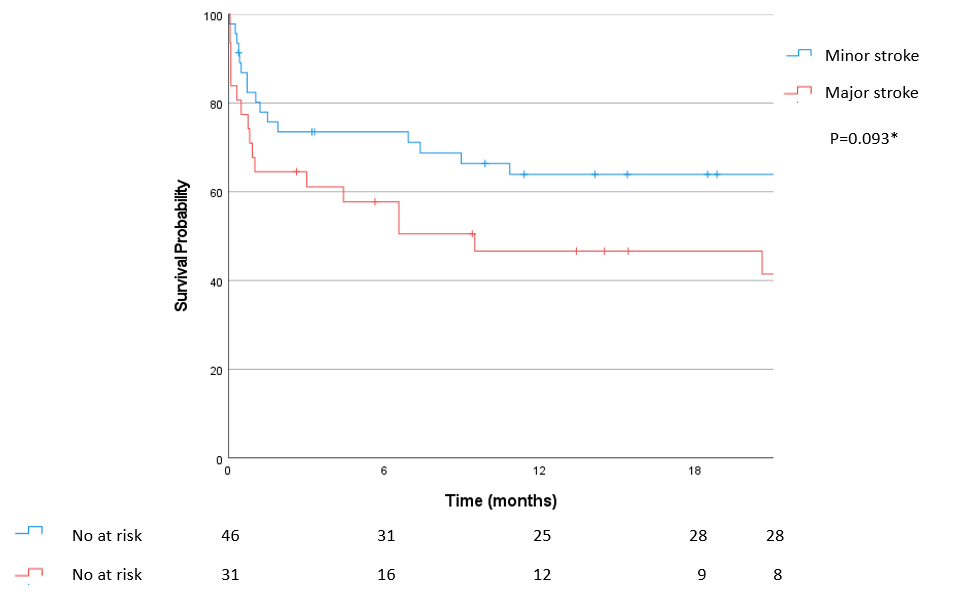**  ***Log rank**  **Supplementary table 1. Logistic regression model with uni- and multivariable risk factor analyses for stroke <30 days.**   \| Variable \| **Count*** \| **Univariate analysis** \| \| \| **Multivariate analysis** \| \| \| \| --- \| --- \| --- \| --- \| --- \| --- \| --- \| --- \| \|  \| % (n) \| OR \| 95% CI \| P \| OR \| 95% CI \| P \| \| ***Aortic zone***** \|  \|  \|  \|  \|  \|  \|  \| \| **Zone 6 to11** \| 0.6% (21/3391) \| Reference \| \| \| Reference \| \| \| \| **Zone 0** \| 22% (13/59) \| 53.9 \| 24.2-120.1 \| <0.001 \| 41.6 \| 18.5-93.7 \| <0.001 \| \| **Zone 1** \| 10.3% (4/39) \| 17.6 \| 4.9-63.0 \| <0.001 \| 16.7 \| 5.2-54.0 \| <0.001 \| \| **Zone 2** \| 7.4% (14/190) \| 11.7 \| 5.2-26.2 \| <0.001 \| 10.6 \| 5.0-22.7 \| <0.001 \| \| **Zone 3** \| 4.4% (11/248) \| 5.2 \| 2.0-13.5 \| <0.001 \| 6.6 \| 3.0-14.6 \| <0.001 \| \| **Zone 4** \| 1.9% (5/270) \| 4.0 \| 1.5-11.0 \| <0.001 \| 2.4 \| 0.9-6.8 \| 0.083 \| \| **Zone 5** \| 1.8% (11/627) \| 2.7 \| 1.2-6.4 \| <0.001 \| 3.1 \| 1.5-6.6 \| 0.003 \| \| **Female sex** \| 3.2% (34/1062) \| 3.0 \| 1.8-5.0 \| <0.001 \| 1.6 \| 1.0-2.6 \| 0.053 \| \| **Age** \|  \|  \| \| \| \| \| \| \| **≤70** \| 1.2% (14/1154) \| Reference \| \| \| Reference \| \| \| \| **71-79** \| 2.1% (50/2343) \| 1.6 \| 0.8-3.2 \| 0.16 \| 2.3 \| 1.2-4.3 \| 0.010 \| \| **≥80** \| 1.1% (15/1345) \| 1.0 \| 0.5-2.3 \| 0.97 \| 1.5 \| 0.7-3.4 \| 0.290 \| \| ***Op. urgency*** \|  \|  \|  \|  \|  \|  \|  \| \| **Elective** \| 1.1% (41/3685) \| Reference \| \| \| Reference \| \| \| \| **Subacute** \| 3.0% (8/267) \| 2.6 \| 1.1-6.3 \| 0.03 \| 2.1 \| 0.9-4.8 \| 0.075 \| \| **Acute** \| 3.4% (30/890) \| 2.9 \| 1.7-5.0 \| <0.001 \| 2.5 \| 1.5-4.4 \| <0.001 \| \| **Prior CVL** \| 3.5% (21/595) \| 4.4 \| 2.7-7.4 \| <0.001 \| 4.4 \| 2.7-7.2 \| <0.001 \| \| **Pathology** \|  \|  \| \| \| \| \| \| \| **Aneurysm** \| 1.4% (61/4512) \| Reference \| \| \|  \| \| \| \| **Dissection** \| 5.5% (18/330) \| 3.9 \| 2.1-7.3 \| <0.001 \|  \|  \|  \| \| **Smoking** \|  \|  \|  \|  \|  \|  \|  \| \| **Never** \| 1.4% (10/694) \| Reference \| \| \|  \|  \|  \| \| **Prior** \| 1.2% (26/2168) \| 0.8 \| 0.7-1.9 \| 0.67 \|  \|  \|  \| \| **Active** \| 1.4% (12/832) \| 1.0 \| 0.4-2.7 \| 0.93 \|  \|  \|  \| \| **Hypertension** \| 1.5% (58/3843) \| 0.7 \| 0.4-1.2 \| 0.21 \|  \|  \|  \| \| **Cardiac risk** \| 1.7% (34/2017) \| 1.1 \| 0.6-1.8 \| 0.85 \|  \|  \|  \| \| **Diabetes** \| 1.1% (8/738) \| 0.7 \| 0.3-1.6 \| 0.43 \|  \|  \|  \| \| **Renal insufficiency** \| 1.4% (10/694) \| 0.9 \| 0.4-1.9 \| 0.78 \|  \|  \|  \|   **Number of strokes within each category. Missing values make the sum differ from 100%.*  ***Aortic Zone: aortic zone for proximal sealing.* Abbreviations: *OR: Odds ratio, CI: Confidence interval, AA:Aortic aneurysm, CVL:Cerebrovascular lesion*  **Supplementary Table 2. A multivariable Cox regression analysis on mid-term survival in regards to perioperative stroke**   \| **Covariate** \| **Hazard ratio** \| **95% Confidence interal** \| **p** \| \| --- \| --- \| --- \| --- \| \| **Age** \|  \| \| \| \| **≤70**  **71-79**  **≥80** \| Reference \| \| \| \| 1.9 \| 1.46-2.46 \| <0.001 \| \| 3.3 \| 2.53-4.38 \| <0.001 \| \| **Female** \| 1.1 \| 0.91-1.35 \| 0.321 \| \| **Hypertension** \| 0.7 \| 0.57-0.87 \| <0.001 \| \| **Diabetes** \| 1.3 \| 1.01-1.58 \| 0.040 \| \| **Cardiac risk** \| 1.3 \| 1.07-1.52 \| 0.008 \| \| **Chronic pulmonary disease** \| 1.6 \| 1.31-1.86 \| <0.001 \| \| **Renal insufficiency** \| 1.8 \| 1.46-2.22 \| <0.001 \| \| **Smoking** \|  \| \| \| \| **Never**  **Prior**  **Active** \| Reference \| \| \| \| 1.1 \| 0.90-1.46 \| 0.281 \| \| 1.4 \| 1.08-1.89 \| 0.013 \| \| **Aortic dissection** \| 0.7 \| 0.44-1.21 \| 0.217 \| \| **Proximal aortic landing zone** \|  \| \| \| \| **Zone 6-11**  **Zone 0**  **Zone 1** \| Reference \| \| \| \| 2.4 \| 1.16-4.89 \| 0.018 \| \| 1.9 \| 0.76-4.62 \| 0.174 \| \| **Zone 2** \| 1.1 \| 0.63-2.04 \| 0.684 \| \| **Zone 3** \| 2.3 \| 1.63-3.36 \| <0.001 \| \| **Zone 4** \| 1.4 \| 0.97-2.12 \| 0.075 \| \| **Zone 5** \| 1.1 \| 0.88-1.48 \| 0.320 \| \| **Urgency of procedure** \|  \| \| \| \| **Elective**  **Sub-acute**  **Acute** \| Reference \| \| \| \| 1.5 \| 1.04-2.06 \| 0.029 \| \| 2.5 \| 2.04-3.14 \| <0.001 \| \| **Perioperative stroke** \| 2.2 \| 1.26-3.72 \| <0.001 \| | *Page 8*  *Page 8*  *Page 8*  *Page 8* |
